# CholeraSeq: a comprehensive genomic pipeline for cholera surveillance and near real-time outbreak investigation

**DOI:** 10.1093/bioinformatics/btaf665

**Published:** 2025-12-16

**Authors:** Massimiliano S Tagliamonte, Abhinav Sharma, Alberto Riva, Monika Moir, Marco Salemi, Cheryl Baxter, Tulio de Oliveira, Carla N Mavian, Eduan Wilkinson

**Affiliations:** Interdisciplinary Center for Biotechnology Research, University of Florida, Gainesville, FL, 32601, United States; DSI-NRF Centre of Excellence for Biomedical Tuberculosis Research, SAMRC Centre for Tuberculosis Research, Division of Molecular Biology and Human Genetics, Faculty of Medicine and Health Sciences, Stellenbosch University, Cape Town, 7505, South Africa; Interdisciplinary Center for Biotechnology Research, University of Florida, Gainesville, FL, 32601, United States; Centre for Epidemic Response and Innovation, School for Data Science and Computational Thinking, Stellenbosch University, Stellenbosch, 7600, South Africa; Emerging Pathogens Institute, Department of Pathology, College of Medicine, Emerging Pathogens Institute, University of Florida, Gainesville, FL, 32601, United States; Centre for Epidemic Response and Innovation, School for Data Science and Computational Thinking, Stellenbosch University, Stellenbosch, 7600, South Africa; Centre for Epidemic Response and Innovation, School for Data Science and Computational Thinking, Stellenbosch University, Stellenbosch, 7600, South Africa; Centre for Epidemic Response and Innovation, School for Data Science and Computational Thinking, Stellenbosch University, Stellenbosch, 7600, South Africa; Emerging Pathogens Institute, Department of Pathology, College of Medicine, Emerging Pathogens Institute, University of Florida, Gainesville, FL, 32601, United States; Centre for Epidemic Response and Innovation, School for Data Science and Computational Thinking, Stellenbosch University, Stellenbosch, 7600, South Africa

## Abstract

**Summary:**

Next Generation Sequencing is widely deployed in cholera-endemic regions, yet an end-to-end reproducible pipeline that unifies read QC, filtering, reference mapping, variant calling/annotation, recombination screening, and extraction of parsimony informative sites/variant codons, phylogenetic inference for downstream phylodynamic and epidemiological analyses have been lacking, slowing outbreak investigation and public health response. CholeraSeq is a high-throughput genomics pipeline for cholera genomic surveillance. It ingests consensus genomes, short read sequence data, draft assemblies, and scales seamlessly from local to cloud environments. To accelerate epidemiological context placement of new outbreak strains, we provide a curated ready-to-use core genome alignment compiled from public data, enabling flexible, fast, integration of new samples for outbreak investigations.

**Availability and implementation:**

CholeraSeq is freely available on the GitHub platform https://github.com/CERI-KRISP/CholeraSeq. CholeraSeq is implemented in Nextflow with a modular design building upon the nf-core community standards.

## 1 Introduction

Whole genome sequencing (WGS) has become essential for epidemiology and outbreak investigations, enabling fine-scale lineage resolution (Achtman 2012, Challacombe *et al.* 2017) and insights into transmission, adaptation, and antimicrobial resistance (Mavian *et al.* 2020, Alam *et al.* 2022, Chabuka *et al.* 2023, Lassalle *et al.* 2023, Mavian *et al.* 2023). *Vibrio cholerae* O1, the agent of cholera and the ongoing seventh pandemic (Kaper *et al.* 1995, Lantagne *et al.* 2014, Domman *et al.* 2017, Weill *et al.* 2017), now has extensive public sequencing data. As of 25 August 2025, there are 3503 Biosamples, and 1307 and 5003 assemblies for *V. cholerae* in Genbank and VibrioWatch (https://pathogen.watch/genomes/all?genusId=662&strain=1) databases, respectively. Turning these data into actionable evidence can be slow, particularly in resource-limited areas (Krampis 2022) and is often hampered by tool/version inconsistencies (Grüning *et al.* 2018, Wratten *et al.* 2021, Cokelaer *et al.* 2023). To address this, CholeraSeq, built on our genomic surveillance experience (Chabuka *et al.* 2023, Mavian *et al.* 2023), is an automated, *V. cholerae*-specific Nextflow pipeline that processes WGS outbreak data from raw reads to high-quality SNPs and phylogenies in near-real-time to support public health efforts.

## 2 Materials and methods

### 2.1 CholeraSeq main workflow

**Figure 1.**
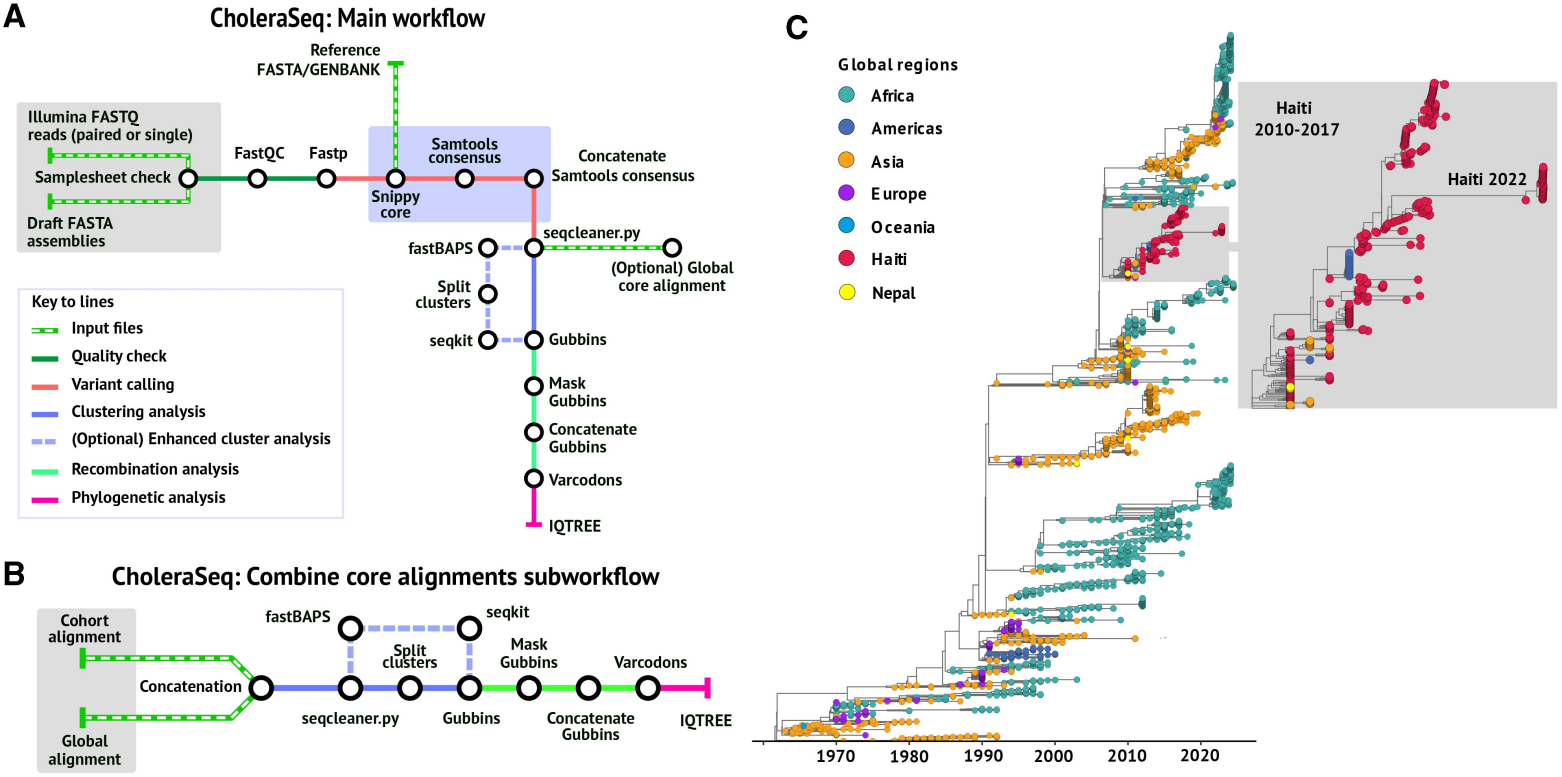
CholeraSeq workflow overview and cholera case study. (A) CholeraSeq main workflow. Raw reads and contigs are processed through quality control (raw reads only), mapped to the reference genome, and are used to generate high-quality SNPs and a consensus genome alignment. Recombination analysis is then performed on this alignment, after which variant positions are filtered and passed to the phylogenetic analysis step. (B) Optional sub-workflow for combining core alignments. New samples can be incorporated into a pre-existing dataset by mapping them to the reference genome and merging the resulting alignments, thereby avoiding the escalation of time and memory requirements each time new data become available. (C) Global *V. cholerae* phylogeny obtained from CholeraSeq and visualized using the R package ggtree. The tree highlights the 2010–2017 and 2022 Haiti clades. Each tip is represented by a circle colored according to geographic origin: Africa, Americas, Asia, Europe, and Oceania. Strains from Haiti, Mexico, Nepal, and Asia clustering within the Haiti lineage are shown with distinct colors for clarity. Branches correspond to units of calendar time (year).

The main workflow of CholeraSeq is shown in Fig. 1A, and a detailed description of the pipeline’s customizable parameters is available here: https://ceri-krisp.github.io/CholeraSeq/parameters.html.

#### 2.1.1 Input data

The CholeraSeq pipeline, in its most basic usage, requires a samplesheet (CSV) that specifies the file paths to input the FASTA/FASTQ files and the sample identifiers to be used in the outputs. The pipeline validates this samplesheet by normalizing sample names (replacing spaces with underscores), verifying accepted file suffixes (e.g. .fq.gz, .fastq.gz), and confirming the presence and accessibility of the listed files in local or cloud storage (including both mates for paired-end data) to prevent runtime failures. Inputs may be raw reads in FASTQ format (single-end or paired-end) or genome assemblies in FASTA format; only FASTQ inputs undergo adapter trimming. Raw long reads (PACBIO and ONT) are not currently supported as input. However, long-read datasets assembled into contigs and supplied as genome assemblies in FASTA format, either alone or in combination with short-read data, are supported.

#### 2.1.2 Quality control

FASTQ raw read quality is assessed with FastQC (https://github.com/s-andrews/FastQC). Quality and trimming statistics are compiled with multiQC (Ewels *et al.* 2016). Quality and adapter trimming is performed with fastp (Chen *et al.* 2018). We selected this tool for its performance, flexibility, and automatic detection of adapter sequences, which streamlines processing of data generated across different sequencing centers and public health laboratories. Our default parameters are: fastp quality trimming with a 4-bp sliding window at a minimum Phred score of 20 (≈1% error rate) and a minimum read length of 35 bp. These thresholds, which remain user-customizable, are intended to account for the inclusion of historical samples generated when Illumina sequencing technology did not yet have the performance and accuracy it offers today. Settings can be modified as needed by adjusting the parameters in either the YAML file or the Nextflow configuration file for advanced configuration.

#### 2.1.3 Alignment and variant calling

The cholera reference genome bundled with our pipeline is the *V. cholerae* strain N16961 (chromosomes accession numbers: NZ_CP028827.1 and NZ_CP028828.1) (Matthey *et al.* 2019) which is widely adopted as the standard reference for *V. cholerae* O1 isolates. Snippy (https://github.com/tseemann/snippy) is used for reference-based alignment and variant calling due to its ability to handle heterogeneous inputs, including contigs/assemblies, single-end reads, and paired-end reads. Snippy consolidates multiple steps, from read mapping and variant calling to functional annotation with SnpEff (Cingolani *et al.* 2012). The consensus genome alignment is produced with SAMtools, this tool was chosen to avoid imputing reference bases at sites with insufficient evidence. The default parameters for this step are minimum base quality = 20; minimum read mapping quality = 20; minimum coverage = 5×; minimum fraction of bases agreeing on the most likely allele = 0.75. Sites failing any criterion were masked as “N.” The relatively permissive coverage threshold (5×) facilitates inclusion of informative low-coverage samples, while the remaining filters reduce spurious genotype calls arising from read misalignment, within-host mixtures, or stochastic sequencing errors at low depth. After generating the consensus alignment, isolates with an excessive proportion of undetermined sites (“N”) and gaps are filtered using seq_cleaner.py with a default threshold of 50%. This relatively permissive cutoff is intended to retain informative low-coverage samples, as bacterial whole-genome alignments typically preserve sufficient variation for robust phylogenetic inference. The threshold is user-configurable.

#### 2.1.4 Clustering and recombination analysis

Majority of bacteria generate genomic variation through substantial horizontal transfer (Arnold *et al.* 2022). In *V. cholerae*, recombination promotes environmental persistence, increases diversity (Keymer and Boehm 2011), and facilitates the transmission of pathogenicity islands (Labbate *et al.* 2016). Accurate identification of horizontally transferred segments is fundamental to robust phylogenetic inference and outbreak source attribution. To this end, our workflow incorporates Gubbins (Croucher *et al.* 2015) to detect putative recombinant regions and remove them from the whole genome alignment. Because deep divergent lineages can mimic the recombination signal and yield false positives, users may optionally perform a clustering step with fastBAPS (Tonkin-Hill *et al.* 2019) and run Gubbins per cluster; clusters with fewer than four sequences are excluded from subsequent analyses due to Gubbins’ tree-estimation requirements. We recommend that the decision to apply clustering be guided by epidemiological context (e.g. inclusion of historical samples from distinct outbreaks) to minimize spurious recombination calls and unnecessary loss of informative sites.

#### 2.1.5 Phylogenetics-ready alignment

After removing recombinant segments, the whole genome alignment can be optionally processed with the built-in varcodons.py tool to reduce computational burden prior to phylogenetic analysis. varcodons.py derives a parsimony-informative sites (PIS) alignment from the full alignment. By default, a site is retained if ≥70% of samples are genotyped (i.e. non-missing), an empirically chosen completeness threshold that preserves broadly defined sites while limiting sparsity; this parameter is user-configurable. The resulting PIS alignment can be used not only for phylogenetics but also for downstream tasks outside CholeraSeq, such as computing SNP distance matrices and assessing intra-/inter-group diversity. In addition, varcodons.py produces a variant-codon alignment (when coding annotation is available) suitable for selection analyses and generates reports detailing the genomic coordinates of SNPs and variant codons, their gene context, and associated annotations.

#### 2.1.6 Phylogenetic inference

The PIS alignment is used to infer a maximum likelihood (ML) phylogenetic tree. By default, the pipeline constructs a ML tree using IQ-TREE (v2.2.0) (Minh *et al.* 2020) with auto nucleotide substitution detection method, which explores all available nucleotide substitution methods implemented in IQ-TREE, and ascertainment bias correction (+ASC) model (Lewis 2001) specific for SNP data. Additionally, it performs 1000 UFBoot replicates to assess support for branches in the phylogeny (Hoang *et al.* 2018).

### 2.2 Optional “combine core alignments” sub-workflow

For users whose primary goal is the rapid investigation of the origin of new strains during an outbreak, we have designed an optional “combine core alignments” sub-workflow (Fig. 1B), which enables the generation of a maximum likelihood tree that situates newly sequenced strains within the global *V. cholerae* context. To this end, we curated a ready-to-use reference core-genome alignment comprising 4196 publicly available *V. cholerae* strains collected worldwide between 1961 and 2024. The input to this sub-workflow consists of two alignments: (i) the cohort core alignment (user outbreak data generated as the output of the main CholeraSeq workflow) and (ii) either the global core alignment available at https://doi.org/10.5281/zenodo.16909942 or a user-specific alignment generated with the same N1696 reference used by CholeraSeq. The sub-workflow concatenates the two input alignments to generate a new core alignment, which is then processed using the same steps as the main workflow (Fig. 1A and B). During an outbreak, data are not always available simultaneously. This sub-workflow allows users to save time and computational resources by efficiently incorporating newly available data during an outbreak investigation.

### 2.3 Software and data availability

The CholeraSeq pipeline is freely available at https://github.com/CERI-KRISP/CholeraSeq. The ready-to-use reference core alignment of 4196 *V. cholerae* O1 genomes available in SRA, ENA, Genbank and VibrioWatch as of 25 August 2025, has been published in Zenodo (https://doi.org/10.5281/zenodo.16909942) along with associated metadata (Table 1, available as supplementary data at *Bioinformatics* online) and will be updated on a quarterly basis. The raw SRA sequence data used to obtain this alignment has a data footprint of ∼1.7Tb, underscoring how this pipeline facilitates quick outbreak investigation using the CholeraSeq sub-workflow. The pipeline codebase is available as an archive on Zenodo: https://doi.org/10.5281/zenodo.15167441, in addition to its Github repository https://github.com/CERI-KRISP/CholeraSeq. The pipeline documentation, including installation, usage and parameters is available on GitHub: https://ceri-krisp.github.io/CholeraSeq/usage.html together with the Github repository.

### 2.4 Portability and resources required by the pipeline

CholeraSeq is designed to make pipeline behavior explicit while enforcing reproducibility and portability via the Nextflow workflow manager (Di Tommaso *et al.* 2017). To standardize its development and maintenance, we adopted the nf-core template (Ewels *et al.* 2020), ensuring consistent structure, best practices, and community review in the Nextflow language. The individual components of the pipeline, such as Gubbins and Snippy are encapsulated as modular components that can be independently updated and tested, facilitating long-term sustainability. The nf-core modules structure further enhances portability through unit tests and prebuilt containers and conda packages, which are activated according to the available computing environment and user provided configuration. Operationally, by default, the pipeline retries failed processes with incrementally adjusted CPU and memory requests accommodating diverse computing infrastructure from laptops to high-performance computing clusters or cloud platforms. When installed locally, all the components of the pipeline can be executed without external internet access, making CholeraSeq suitable for deployments with stringent data-governance or confidentiality requirements.

### 2.5 Pipeline usability: a case study of Haiti 2022 outbreak

To evaluate the pipeline’s usability and performance we analyzed the dataset from BioProject PRJNA900632, which documents the re-emergence of cholera in Haiti in 2022 (Mavian *et al.* 2023). We downloaded the 41 strains (BioProject PRJNA900632), processed them with the main CholeraSeq workflow (Fig. 1A), and merged the output core alignment with the ready-to-use core-alignment using the optional sub-workflow (Fig. 1B). A timescaled phylogeny was inferred with TreeTime (0.11.0) (Sagulenko *et al.* 2018) using the ML tree produced by CholeraSeq. Tree visualization was performed in R with the ggtree package (Yu *et al.* 2017). TreeTime and R visualization are not currently supported within CholeraSeq, however are planned for inclusion in a future release driven by user-feedback.

## 3 Results

### 3.1 Phylogenetics-ready alignment

The final alignment produced by the pipeline comprises PIS extracted from the recombination-masked core genome alignment. High-quality single nucleotide substitutions were called relative to the *V. cholerae* N16961 reference. In total, 5897 parsimony informative sites were extracted from 4196 taxa with 2215 genomes from Africa and 1275 from Asia. The temporal sampling frame of this alignment spans 1961–2024 capturing over six decades of *V. cholerae* O1 circulation during the ongoing 7th pandemic.

### 3.2 Case-study and interpretation of results

As a case-study we repeated the outbreak investigation of the 2022 cholera outbreak in Haiti (Mavian *et al.* 2023). This outbreak investigation uses both CholeraSeq workflows: Firstly, we process the 41 strains through the main CholeraSeq workflow to obtain a core genome alignment (Fig. 1A). Secondly, we use the optional sub-workflow to merge the outbreak core alignment with 4196 global cholera genomes using the ready-to-use core alignment provided by CholeraSeq and obtain a worldwide phylogeny (Fig. 1B). The run was executed on a Google Cloud server equipped with 32 CPUs and 250 GB RAM and completed in 1 h and 25 min. Our new global phylogenetic tree composed of 4237 isolates, including the 41 strains from 2022 and 327 strains from Haiti sampled between 2010 and 2019, confirmed previous findings that the recent cholera outbreak was closely related to strains that had circulated in Haiti during earlier years (Fig. 1C).

## 4 Discussion

We present CholeraSeq, an automated workflow that integrates established genomics tools to support epidemiological investigations of cholera outbreaks in academic and public health settings. The pipeline is designed to deliver near real-time, actionable insights by processing newly generated genomic data and placing them within the phylogenetic context of prior studies, as soon as outbreak data is available. The pipeline accommodates single-end, paired-end reads, and FASTA assemblies; combines published whole-genome datasets across formats; and supports iterative updates so new sequences can be appended to existing datasets without re-analyzing the entire dataset. Users may also annotate and extract variant codons to enable downstream selection analyses (e.g. synonymous versus non-synonymous changes).

Although designed for *V. cholerae* O1 outbreaks, CholeraSeq is adaptable to other *Vibrio* species or bacterial organisms by supplying an alternative FASTA/GenBank format reference, enabling SnpEff based VCF annotation (Cingolani *et al.* 2012). Because it is reference-based, the pipeline mitigates misalignment artifacts common in pan-genome analyses; however comprehensive pan-genome studies would require *de-novo* assembly, which is not currently included. Consequently, the resulting core-genome alignment excludes insertions and genes absent from the reference and is not suited to surveying mobile elements or resistance determinants outside the core genome. BAM files produced as intermediates can nonetheless be repurposed for such analyses and may inform future expansion modules based on user feedback.

In summary, we provide an integrated genomic workflow for outbreak tracking, taking the user from raw sequencing data to the output phylogenetic tree. The whole pipeline is implemented with Nextflow, which facilitates portability across platforms, parallelization, and replicability of results. Compared to other pipelines that process bacterial genomic data, such as bacpage (https://github.com/CholGen/bacpage) and Bactopia (Petit and Read 2020), CholeraSeq provides an end-to-end workflow that eliminates manual intervention, particularly when handling heterogeneous inputs. In this respect, CholeraSeq is conceptually more similar to nf-core/bactmap (Ewels *et al.* 2020) with two key differences: (i) it natively accepts multiple input formats within a single run, and (ii) it includes a built-in option to process new samples and append them to a previously generated alignment, thereby minimizing runtime for iterative updates. Because the pipeline is tailored primarily for cholera outbreaks investigations, users can leverage the ready-to-use global core genome alignment distributed with CholeraSeq and updated on a quarterly basis to rapidly place their isolates within a worldwide cholera lineage context. Additionally, CholeraSeq outputs a variant codon alignment that users can use for downstream evolutionary and selection analyses.

CholeraSeq is a user-friendly pipeline for cholera outbreak investigation, designed to minimize computational burden and remain accessible in low- and middle-income country settings, including for users with limited bioinformatics experience.

## Supplementary Material

btaf665_Supplementary_Data

## Data Availability

CholeraSeq is freely available on the GitHub platform https://github.com/CERI-KRISP/CholeraSeq
